# Pyridostigmine induced heart block requiring ICU admission

**DOI:** 10.1080/20009666.2018.1527668

**Published:** 2018-10-15

**Authors:** Benjamin Chaucer, Dustin Whelan, Dronacharya Lamichhane

**Affiliations:** a University of Illinois College of Medicine at Peoria, Peoria, IL, USA; b OSF HealthCare-Illinois Neurological Institute, University of Illinois College of Medicine-Peoria

**Keywords:** Heart block, pyridostigmine, ICU, EKG, myasthenia gravis

## Abstract

Myasthenia gravis is an autoimmune disorder that effects an estimated 20 people per 100,000 in the USA per year. Pyridostigmine is a common drug used in the symptomatic treatment of myasthenia gravis. While generally safe and effective, a rare set of patients treated with pyridostigmine encounter cardiac conduction disorders. Here, we report a rare presentation of an adverse drug reaction due to pyridostigmine, which is important for its implications in the acute care setting.

## 1. Introduction

Myasthenia Gravis (MG) is an autoimmune disorder characterized by autoantibodies to the acetylcholine receptors at the neuromuscular junction of skeletal muscles. The hallmark of the illness is fluctuating muscular weakness in voluntary skeletal muscle. Pyridostigmine is a common symptomatic treatment for MG. It is a well-studied and generally safe treatment for myasthenia gravis that works by inhibiting acetylcholinesterase, the enzyme responsible for breaking down acetylcholine at the neuromuscular junction. A rare complication of pyridostigmine is heart block. This is thought to be secondary to muscarinic effects of acetylcholine on cardiac tissue. We present a case of severe symptomatic bradycardia and third degree AV block secondary to pyridostigmine.

## 2. Case report

The patient is a 73-year-old female with past medical history of sero-positive ocular Myasthenia Gravis (with anti-acetylcholine receptor antibody) and COPD who presented by helicopter with altered mental status and weakness. On presentation the patient was hypotensive and afebrile with a heart rate in the 20s. She received atropine en route and was started on a dopamine drip along with aggressive fluid resuscitation. Blood pressure improved to 111/54 with HR increasing to the low 30’s. Lab work at outside hospital showed sodium 145 mmol/L, potassium 6.0 mmol/L, chloride 107 mmol/L, with a BUN of 41 mg/dL and creatinine 2.32 mg/dL. POC glucose was 191 and AST 55 U/L with ALT 37 U/L. Her hyperkalemia was treated with insulin and dextrose and had returned to normal limits by time of presentation. Repeat kidney, liver function tests, and serum electrolytes were within normal limits. Troponins were drawn and found to be 0.062 ng/mL with a Brain Natriuretic Peptide of 60 pg/ml. ECG was performed and showed patient had a complete heart block. The patient was admitted to Medical Intensive Care Unit where transcutaneous pacing was attempted but found to be ineffective. The patient was brought to the cardiac cath lab for transvenous pacing. Review of prior to admission medications showed that the patient had taken her PO pyridostigmine prior to developing bradycardia with altered mental status. She denied taking any of her COPD medication prior to presentation including any short or long acting beta agonists. Neurology was consulted for further evaluation of causes of heart block in a patient with MG. Pyridostigmine was held for concern of its affect in leading to complete heart block. With pyridostigmine held, the patient reverted back to sinus rhythm and transvenous pacing was removed. Using the Naranjo scale for adverse drug reaction patient received a score of 6 for a probable adverse drug reaction. Given the patient’s lack of infectious etiology, including negative Lyme serology, alternative pharmacological causes of heart block and reversion to sinus rhythm after removal of the offending agent, pyridostigmine was diagnosed as the causative agent. When the patient was stabilized she was discharged home with pyridostigmine held with close follow-up with her neurologist for further management of her MG.

## 3. Discussion

MG is an autoimmune disease of the neuromuscular junction. Autoantibodies attack the acetylcholine receptors therefore decreasing the effect of acetylcholine at the neuromuscular junction [1]. Treatment is aimed at overcoming the effects of the autoantibodies by increasing the amount of acetylcholine at the postsynaptic junction. One common and well-studied treatment for MG is pyridostigmine. Pyridostigmine is an acetylcholinesterase inhibitor that decreases the break- down of acetylcholine at the post synaptic junction. This results in an increase in the amount of acetylcholine available for the already depleted acetylcholine receptors. Acetylcholine’s effects are not limited to the skeletal muscle. As many as 12% of patients who suffer from MG experience clinical signs of cardiac disease [2]. The incidence of clinical signs of cardiac disease increases to 50% in patients with MG who have a concomitant thymoma [2]. In some subtypes of MG, patients have striational antibodies that bind to skeletal muscle and to titin in cardiac tissue [3]. Myocarditis has been documented in as much as 35% of the patients with MG with anti-striational antibodies [4]. In patients with MG and a thymoma, incidence of anti-striational antibodies are as high as 97 % [5]. To date several case reports of pyridostigmine-induced bradycardia and heart block, have been documented in current literature [6–9]. One study performed in 1971 on prepared cardiac tissue showed that acetylcholine decreased the cyclic AMP released by stimulation of adrenaline therefore slowing conduction rate [10]. Recent case reports demonstrate that anticholinesterase inhibitors like pyridostigmine have resulted in bradyarrythmias and AV blocks often requiring pacemaker placement or treatment with hyoscyamine [6]. The effects of acetylcholine on the heart are mediated by muscarinic (M2) receptors on cardiac nodal and atrial tissue. This muscarinic reaction leads to increased sodium permeability. The M2 receptors are abundant in nodal tissue and the binding of acetylcholine to these receptors leads to slowing of the heart rate [10]. In our patient removal of the offending agent was sufficient to reverse the effects of the heart block. Our patient avoided long term pacemaker placement by stopping treatment of pyridostigmine. Whether the resulting heart block is due directly to pyridostigmine or a secondary effect of increased acetylcholine is not known. However, removal of pyridostigmine in patients with MG who experience AV block may serve as an initial conservative step in the treatment of pyridostigmine-induced AV block and bradyarrhythmias in the acute care setting. In patients with previous history of MG who present with heart block and bradyarrhythmias currently being treated with pyridostigmine, medication-induced arrhythmia should be considered.

## References

[1] Kasper D, Fauci A, Hauser S, et al Harrison’s principles of internal medicine. NewYork: McGraw Hill; 2015.

[2] Guglin M, Campellone JV, Heintz K, et al Cardiac disease in myasthenia gravis: a literature review. J Clin Neuromuscul Dis. 2003;4(4):199–203. 19078714 10.1097/00131402-200306000-00006

[3] Romi F, Skeie GO, Gilhus NE, et al Striational antibodies in myasthenia gravis: reactivity and possible clinical significance. Arch Neurol. 2005;62(3):442–446. 15767509 10.1001/archneur.62.3.442

[4] Suzuki S, Utsugisawa K, Yoshikawa H, et al Autoimmune targets of heart and skeletal muscles in myasthenia gravis. Arch Neurol. 2009;66:1334–1338. 19752287 10.1001/archneurol.2009.229

[5] Shivamurthy P, Parker MW. Cardiac manifestations of myasthenia gravis: a systematic review. IJC Metab Endocr. 2014;5:3–6.

[6] Pyridostigmine induced prolonged asystole in a patient with myasthenia gravis successfully treated with hyoscyamine Case Reports in Cardiology. 2017;2017 DOI 10.1155/2017/6956298 PMC5446877 28589042

[7] Arsura EL, Brunner NG, Namba T, et al Adverse cardiovascular effects of anticholinesterase medications. Am J Med Sci. 1987;293(1):18–23. 3812546 10.1097/00000441-198701000-00005

[8] Gehi MB, Langberg J Treatment of pyridostigmine-induced AV block with hyoscyamine in a patient with myasthenia gravis. J Cardiovasc Electrophysiol. 2008;19(2):214–216. 17711430 10.1111/j.1540-8167.2007.00938.x

[9] Said S, Cooper CJ, Alkhateeb H, et al Pyridostigmine-induced high grade SA-block in a patient with myasthenia gravis. Am J Case Rep. 2013;14:359–361. 24046803 10.12659/AJCR.889484 PMC3775617

[10] Lee TP, Kuo JF, Greengard P Regulation of myocardial cyclic AMP by isopro- terenol, glucagon and acetylcholine. Biochem Biophys Res Con. 1971;45:991–997. 10.1016/0006-291x(71)90435-9 4330147

